# Alveolar Soft Part Sarcoma with Unusual Cardiac Metastasis: A Case Report and Review of the Literature

**DOI:** 10.1155/2017/7248727

**Published:** 2017-08-06

**Authors:** Abhinav Tiwari, Bhavana Siddegowda Bangalore, Himani Sharma, Zaid Ammari, Mohammad S. Khan, Zubair Khan, Hermann Simo

**Affiliations:** ^1^Department of Internal Medicine, University of Toledo Medical Center, 3000 Arlington Avenue, MS 1150, Toledo, OH 43614, USA; ^2^Department of Cardiology, University of Toledo Medical Center, 3000 Arlington Avenue, MS 1150, Toledo, OH 43614, USA

## Abstract

Alveolar soft part sarcoma is a very uncommon soft tissue malignancy which accounts for <1% of soft tissue sarcoma. It is a malignant and highly vascular tumor arising most commonly in the musculature of the lower extremities, with metastasis primarily to the lungs, bones, and brain. Cardiac metastasis is very rare and only 5 cases have been reported in the literature so far. We report a case of a young woman with a history of surgically resected alveolar soft part sarcoma of left thigh who presented with persistent dry cough and was found to have a cardiac mass, which on biopsy proved to be alveolar soft part sarcoma.

## 1. Introduction

Alveolar soft part sarcoma (ASPS) is a rare soft tissue neoplasm that accounts for less than 1% of all soft tissue sarcomas [1] and affects individuals between 15 and 35 years of age primarily, with a slight female preponderance [2]. In adults, the most common site is the deep soft tissues in the thigh or buttock. However, it is known to occur in organs such as lungs, breasts, stomach, female genital organs, and bones [3, 4]. It is a malignant and highly vascular tumor, and metastases to the lungs, bones, and brain are common while cardiac metastasis is extremely rarely [5, 6]. We report a case of a young woman who had history of ASPS of the left thigh which was resected. She presented one year later with a dry cough and was found to have a cardiac mass, which on biopsy was consistent with ASPS.

## 2. Case Presentation

**Figure 1:**
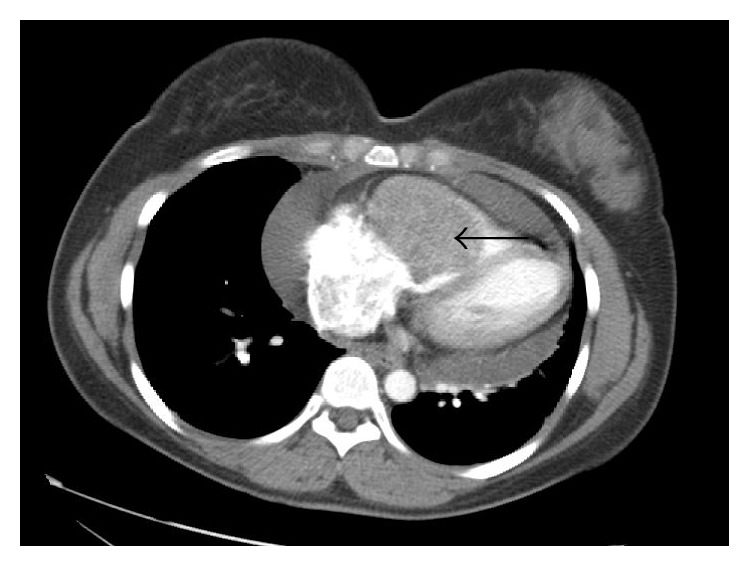
Computerized tomography of chest with contrast showing a large right ventricular mass (arrow) and pericardial effusion.

**Figure 2:**
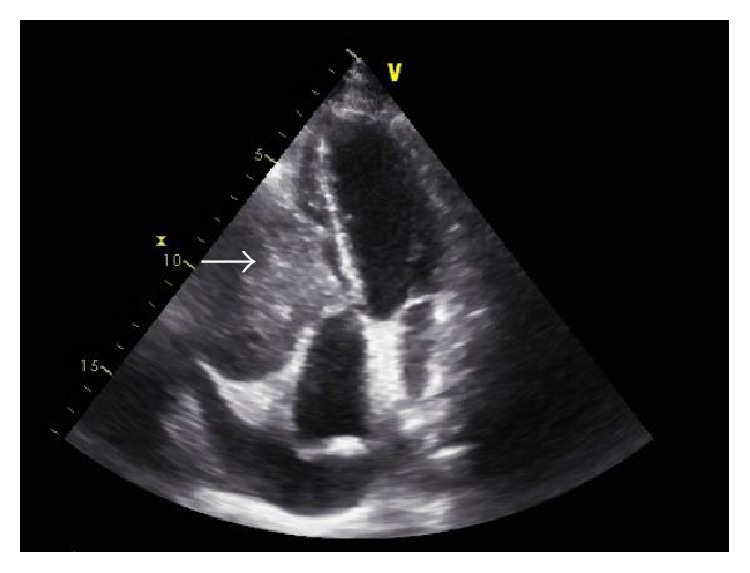
2D echocardiogram of the heart in apical four chamber view showing a right ventricular mass (arrow) arising from the free wall.

**Figure 3:**
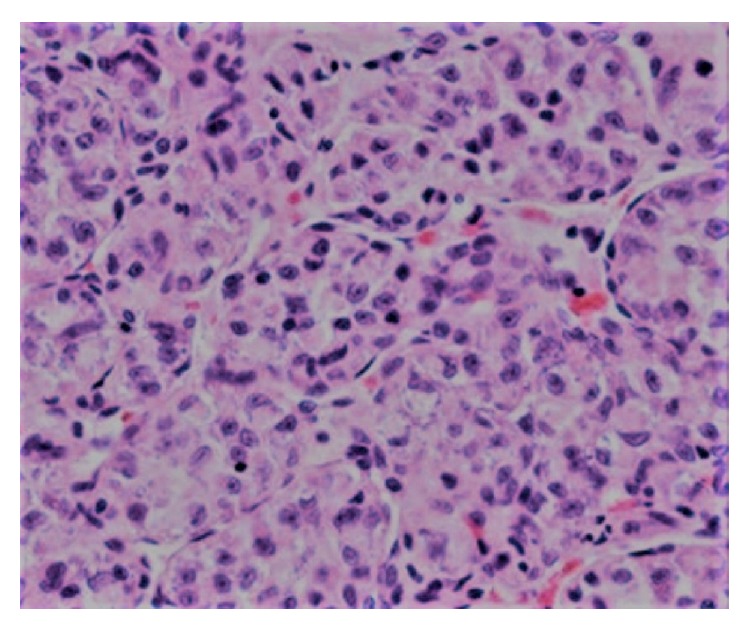
Hematoxylin-eosin, original magnification ×400. The figure shows large polygonal tumor cells in nests with eosinophilic cytoplasm and central vesicular nuclei with prominent nucleoli.

A 31-year-old female with a history of ASPS in the left thigh treated with neoadjuvant radiation followed by radical excision of the mass was seen one year later for dry cough of 1-month duration. She denied any chest pain, fever, or shortness of breath. Cough was refractory to over-the-counter cough suppressants. She had a normal chest X-ray and was even treated with a 5-day course of azithromycin by the primary care physician. On exam, she had normal lung sounds while cardiac examination revealed a 2/6 holosystolic murmur over the left heart border. Due to persistent nature of this cough, a computerized tomography (CT) scan of the chest was performed that revealed a large (4.5 cm) right ventricular (RV) mass and pericardial effusion (Figure 1()). She was referred to cardiology clinic, where a transthoracic echocardiogram showed a large right ventricular mass about 5 cm in size. She was then taken for right heart catheterization (RHC) to obtain a tissue specimen. Preoperative 2D echocardiogram confirmed a large 5.5 × 7.5 cm mass that was intimately associated with right ventricular free wall encroaching upon the AV groove and interfering with the tricuspid valve (TV) mechanism (Figure 2()). On RHC, the pressure findings were consistent with the physiology of TV stenosis caused by obstructing mass. Pulmonary capillary wedge pressure was 5 mm Hg, RV pressure was 25/3 mm Hg, the right atrial pressure was 14 mm Hg, and pulmonary artery pressure was 15/6 mm Hg. Pericardiocentesis was performed and 420 mL of hemorrhagic pericardial fluid was drained. Intraoperative frozen section of the RV mass was consistent with ASPS (Figure 3()). Transcription factor E3 (TFE-3) immunostain revealed strong and diffuse nuclear immunoreactivity of tumor cells. The patient was transferred to a specialized cardiothoracic surgical center for intervention, where partial resection of the cardiac mass was performed.

## 3. Discussion

ASPS is a very uncommon soft tissue malignancy that was originally described by Smetana and Scott Jr. in 1951 as a malignant tumor of the nonchromaffin paraganglia [7]. Christopherson et al. later described it under the name of “alveolar soft part sarcoma” in 1952 [8]. ASPS accounts for <1% of soft tissue sarcoma and typically occurs in young adults with a female predominance [9, 10]. Although the most common site of the tumor is the musculature of the lower extremities [11], it is also known to occur in organs such as lungs, breasts, stomach, female genital organs, and bones [3, 4]. Typically, patients note a painless, slowly growing mass; however, some patients may present primarily with metastatic disease. It is an indolent and slowly growing tumor, but the overall 5-year survival is only 20% for nonresectable tumors [12, 13]. Despite being slow growing, it is a malignant and highly vascular tumor which metastasizes primarily to the lungs, bones, and brain, while cardiac metastasis is extremely rarely [5, 6]. Hematogenous dissemination which can occur long after resection of the primary tumor, even if there is no local recurrence [14–16].

To the best of our knowledge, there have been five cases of cardiac metastases reported so far. Chen et al. reported a case of ASPS of left arm which 3 years later metastasized to heart, brain, lung, and spleen. The patient succumbed to cachexia and biopsy or resection of the cardiac tumor was not performed [17]. Akiyama et al. reported a case of ASPS of the right thigh which was surgically removed and later metastasized to lungs, brain, intestines, and heart [18]. Strecker et al. reported a case of ASPS of right thigh with brain and later cardiac metastases, which could be excised partially [19]. Campbell et al. described a case of surgically resected ASPS of the right forearm which later spread to lungs, heart, and brain. Cardiac lesion was asymptomatic and was not resected [20]. Finally, Stark et al. described a case of left thigh ASPS which was surgically excised; 5 years later, the patient presented with signs of congestive heart failure and was diagnosed with cardiac metastasis of the tumor, which was surgically resected [21].

On imaging, the tumor is contrast enhancing due to its vascular nature [22]. ASPS tend to have high signal intensity on T1- to T2-weighted images on magnetic resonance imaging. On immunohistochemistry, ASPS is consistently positive for an antibody that detects carboxyl terminal of transcription factor 3 (TFE3) gene in the form of strong nuclear staining pattern [23, 24].

Although rarely curative, the cornerstone of treatment remains surgical excision of the primary and isolated metastases [12, 18]. Radiotherapy should be added as a supplemental procedure if there is partial resection of the tumor, positive surgical margin, or presence of symptomatic metastasis. There is a limited role of chemotherapy in treating solitary lesion. However, in the presence of multiple metastases of the lungs, brain, spleen, or heart, chemotherapy may be considered a treatment option. There is a limited success with anthracycline based chemotherapy regimens but the overall prognosis of ASPS is poor. Kummar et al. treated 43 metastatic, unresectable ASPS patients with cediranib (30 mg) once daily in 28-day cycles, and it was observed that cediranib has substantial single-agent activity, producing an overall remission rate of 35% and a disease control rate of 84% at 24 weeks [12]. This trial is still ongoing.

In conclusion, this is a case of ASPS with rare event of cardiac metastasis. The patient with cardiac metastasis can remain asymptomatic or may have subtle symptoms such as cough or dyspnea. Such patients may also present with signs of frank congestive heart failure [21]. Therefore, in a patient with treated or untreated ASPS, it is imperative to consider cardiac metastasis in addition to more common pulmonary metastasis if the patient presents with respiratory or cardiac symptoms.
